# Acceptance of screening for Intimate Partner Violence, actual screening and satisfaction with care amongst female clients visiting a health facility in Kano, Nigeria

**DOI:** 10.4102/phcfm.v3i1.174

**Published:** 2011-07-04

**Authors:** Ime A. John, Stephen Lawoko, Abimbola Oluwatosin

**Affiliations:** 1 Department of Public Health Sciences, Karolinska Institute, Sweden; 2 Department of Nursing, Faculty of Clinical Sciences, College of Medicine, University of Ibadan, Nigeria

## Abstract

**Background:**

Healthcare providers have advocated for the screening and management of Intimate Partner Violence (IPV) against women and its consequences. Unfortunately, data from high income countries suggest that women may have varied preferences for being screened for IPV in healthcare. Although women's preference for screening in sub-Saharan countries has not been well researched, IPV remains an accepted societal norm in many of these countries, including Nigeria.

**Objective:**

The objective of the study was to assess women's acceptance of screening for IPV in healthcare, the extent to which inquiry about IPV was carried out in healthcare and whether such inquiry impacted on satisfaction with care.

**Method:**

Data on these variables were gathered through structured interviews from a sample of 507 women at a regional hospital in Kano, Nigeria. The study design was cross-sectional.

**Results:**

The results found acceptance for screening in the sample to be high (76%), but few women (7%) had actually been probed about violence in their contact with care providers. Acceptance for screening was associated with being married and being employed. Actual screening was associated with ethnicity and religion, where ethnic and religious majorities were more likely to be screened. Finally, being screened for IPV seemed to improve satisfaction with care.

**Conclusion:**

The findings demonstrate the need for adaptation of a screening protocol that is also sensitive to detect IPV amongst all ethnic and religious groups. The findings also have implications for further education of socio-economically disadvantaged women on the benefits of screening.

## Introduction

### Setting

A recent development in the prevention and control of Intimate Partner Violence (IPV) has been the involvement of healthcare professionals through screening for the practice amongst their female clients.^1, 2^ Yet, data from high income countries suggest that women may have varied preferences for being screened for IPV in healthcare.^3, 4, 5, 6^ Although women's preference for screening in sub-Saharan countries has not been well-researched, IPV remains an accepted societal norm in many of these countries including Nigeria.^7, 8, 9, 10, 11, 12, 13, 14, 15^ In fact, women in these settings appear to endorse IPV to a higher degree than the men,^9, 16^ raising questions as to whether female clients in such settings may accept being screened for IPV. Moreover, institutionalisation of IPV seems apparent in many parts of sub-Saharan Africa. In Northern Nigeria for example, the penal code (*Section 55d*) permits a husband to ‘correct’ his wife provided no grievous hurt is inflicted in the process.^17^ Such norms and laws have prompted women to blame themselves for abuse^18^ and may also demotivate them from disclosing abuse.^19^

In other settings outside Africa, women's preferences for screening have been attributed to a number of factors including, shame, fear of retaliation from the husband's relatives and an increased risk for divorce in case the husband finds out.^20, 21, 22, 23,24^ Furthermore, the protection of family honour, and the possible economic and emotional dependence of women on their husbands may further impact on disclosure and thereby on preference for screening amongst women.^20, 25, 26^ Although the role of some sociodemographic variables (e.g. ethnicity, religion, education and literacy) for women's preferences for screening remain elusive, these variables have consistently been reported to affect women's attitudes towards IPV,^27, 28, 29^ suggesting that they might also be related to women's willingness to be screened. This study will undertake to understand the extent to which women in Kano, Nigeria, are willing to be screened for IPV and whether this will vary depending on sociodemographic characteristics. Findings from this study could be useful in identifying demographic barriers to screening amongst clients and inform interventions to improve screening in the study setting.

Unlike in many developed countries, screening for IPV in healthcare is not yet routine practice in most middle and low income countries.^30, 31^ Still, an assessment of provider screening at own initiative may give some idea of readiness for routine screening in healthcare in low income countries. This study aims to assess the extent to which healthcare providers undertake screening on their own initiative in Kano, Nigeria.

Whilst screening for IPV has received significant advocacy during the past two decade,^2, 32^ assessment of the outcome and benefits of screening has not received equivocal attention. One such outcome measure could be *satisfaction with care*. *Satisfaction with care* has recently emerged as an indicator for good medical practice that includes screening for IPV.^31^ Whether or not screening for IPV improves women's satisfaction with different aspects of care deserves further scrutiny. This paper aims to investigate this notion.

### Significance of the study

Based on this background, the current study will assess women's acceptance for screening for IPV in healthcare, the extent to which inquiry about IPV is carried out in healthcare and whether such inquiry impacts on *satisfaction with care*. More concretely, answers to the following research questions will be sought:

- What are women's preferences with regard to screening for IPV in Kano, Nigeria and do these preferences depend on sociodemographic factors?
- How prevalent are inquiries about IPV in healthcare in Kano, Nigeria and does inquiries vary depending on the client's social demographic characteristics?
- Does inquiry about IPV improve a client's *satisfaction with care*?

## Ethical considerations

Ethical approval for this study was obtained from the Nigerian Institute of Medical Research, Lagos, Nigeria and permission was granted by the authorities of Aminu Kano Teaching Hospital, Kano. Participants’ identification information (e.g. names and addresses) were not requested. The aims and relevance of the study were explained to participants when their consent was requested in a separate document accompanying the structured interview format (questionnaire). Voluntary participation was emphasised, privacy guaranteed and informed consent obtained.

## Method

### Setting and materials

Data for this study were collected at the Aminu Kano Teaching Hospital in Kano, Nigeria. Screening was not routinely practised at this centre at the time of our study; therefore if screening was practised at all, it was on individual initiative of the health care providers. Aminu Kano Teaching Hospital is a fully equipped (tertiary) health institution that should provide tertiary health care services; however, the hospital provides primary and secondary care services in some instances. The Departments of Psychiatry and the Social Welfare have experts and personnel to manage victims of Intimate Partner Violence whenever they are referred to them. The study was based on structured interviews with women attending the General Outpatient Department, and the maternal and child health clinics at the hospital. Five female and one male assistant were recruited to assist in the interviews. All the female assistants were nurses or midwives of different grades. The male assistant was a final year medical student. Two training sessions on the study, that is, its aims, questionnaire administration and ethical consideration, were conducted for the assistants.

### Design

The study design was cross-sectional. One in three women was politely approached by one of the assistants as they left the consultation room. The project was explained briefly to the patient and if she consented, she was escorted to an adjoining room where the second assistant was stationed. The procedure and project was explained in more detail and the participant was assured of the confidentiality of the study, as well as reminded that she had the right to withdraw at any stage without any personal repercussion. Every time after an interview was completed, the next woman visiting the clinic would be approached until responses from 507 women were achieved. This sample size was based on a power analysis assuming a binomial distribution with a prevalence of IPV in developing countries of 0.12 estimated from previous studies,^34, 35^ a statistical significance level (*alpha* = 0.05) and a power of 80%. Forty women declined to participate in the study.

### Procedure

Each interview was performed by a pair of interviewers using structured questionnaires. Participants’ *preferences for screening for IPV* were assessed by asking participants to describe how they would feel if asked about abuse in their intimate relationship in a healthcare situation. The concept of abuse was explained to each participant that consented to be interviewed. Then she was asked ‘how she felt if asked question(s) on the subject of IPV against women’. This was an open-ended question. Two assistants previously trained in IPV listened carefully to every respondent and recorded the responses in their notebooks. After each interview the pair of assistants discussed the participant's response to the open-ended question, reached a consensus and placed the result under one of four predetermined options:

- ‘Acceptable’
- ‘Neither acceptable nor unacceptable’
- ‘Both acceptable and unacceptable’
- ‘Unacceptable’.

An *Acceptable* response implied that the participant expressed a clear and positive attitude to being questioned about IPV. A *Neither acceptable nor unacceptable* response implied that the client expressed no sign of being offended by the question, nor any overtly acceptable expression. A *Both acceptable and unacceptable* response implied that the client expressed a positive attitude to being asked about IPV, but also expressed some disagreement to being asked such questions, that is, a mixed response. An *Unacceptable* response by the participants implied offence or discomfort expressed when questioned on IPV. These categories have been determined previously in qualitative studies and responses validated.^3^

*Actual screening* was assessed by asking the interviewed women whether they had been questioned by their health care provider during the present contact about the possibility of IPV. Response alternatives were ‘yes’ or ‘no’.

*Clients’ satisfaction with care* was probed with the *pyramid patient questionnaire*, a previously validated instrument with three subscales.^36^ Nurses’ competence and skills were assessed by the subscale *‘Nursing Staff’* (this subscale consists of three items. Cronbach's alpha for the current sample was 0.87). Contact with the staff was assessed by the subscale *‘Contact’* (this subscale consists of three items; Cronbach's alpha for the current sample was 0.83). Support of the Client was assessed by the subscale *‘Social Support’* (this subscale consists of four items; Cronbach's alpha for the current sample was 0.83). For each item under these subscales, the response alternatives were scored using the Likert's score of 1–4 (1 equalled ‘Not at all’; 2 was ‘No’; 3 was ‘Yes’; and 4 equalled ‘Strong Yes’). High scores, therefore, reflected higher satisfaction both in specific items and subscales.

#### Sociodemographic

Indicators including age, marital status, and the number of children, profession, religion, ethnicity, literacy level, educational level and employment status were recorded and the response alternatives for these variables were noted (Table 1).

**TABLE 1:** Demographic characteristics of participants in screening for Intimate Partner Violence.

Demographic characteristics	Additional characteristics	*n*	%
Profession	Housewife	183	49.1
	Others†	189	50.9
Marital status	Married	373	73.6
	Single	113	22.3
	Divorced or Separated	21	4.1
Religion	Islam	352	69.4
	Others‡	155	30.6
Ethnicity	Hausa or Fulani	316	62.5
	Others§	190	37.5
Children (per woman)	0	159	31.4
	1–2	128	25.2
	3–4	127	25.0
	5 and above	93	18.4
Marital situation	Monogamous	207	62.2
	Polygamous	126	37.8
Employment	Employed	133	26.7
	Not employed	365	73.3
Literacy level	Cannot read at all	82	16.6
	Can read part of sentence	79	16.0
	Able to read whole sentences	334	67.5
Educational level	No education	75	14.9
	Primary	37	7.3
	Secondary	189	37.5
	Post-secondary	199	39.5
	Islamic or Quranic	4	0.8

*Source*: Authors’ original data

*n*, given as absolute number.

† Gainfully employed professionals including students.

‡ Christianity and other minority religion.

§ Yoruba, Ibo and other ethnic groups.

### Analysing

The Chi-square test was used to assess associations between participants’ preferences for screening and sociodemographic variables. There were only a few participants in two categories of the variable measuring acceptability for screening (i.e. ‘*Neither acceptable nor unacceptable’* and ‘*Both acceptable and unacceptable’)* and consequently a dichotomous variable was formed with the other two options, that is ‘*Acceptable’ and ‘Unacceptable’*. Associations between actual screenings for IPV and the different *satisfaction with care* subscales or total satisfaction was assessed using the student *t*-test. Statistical significance was assumed at *p* < 0.05 and SPSS (Statistical Package for the Social Sciences) version 16.0 for Windows was used for all the analyses.

## Results

### Demographic characteristics of participants

The majority of participants were married, Muslims, of Hausa or Fulani ethnic belonging, unemployed, literate and with at least primary school education (Table 1). The average age of the sample was 29 years with a Standard Deviation of eight years.

### Acceptance for Intimate Partner Violence inquiry and actual Intimate Partner Violence inquiry

The majority (76%, *n* = 355) of the participants found it acceptable to be probed about IPV in healthcare and almost 20% regarded such inquiries as unacceptable (Table 2). The healthcare provider had probed 7% of the interviewed women on the possibility of IPV during their latest visit.

**TABLE 2:** Acceptance of Intimate Partner Violence inquiry and actual Intimate Partner Violence inquiry.

Partner Violence inquiry	*n*	%
**Intimate**		
Acceptable	355	76.0
Neither acceptable nor unacceptable	7	1.5
Both acceptable and unacceptable	12	2.6
Unacceptable	93	19.9
**Total**	**467**	**100**
**Actual**		
Yes	33	6.9
No	446	93.1

*Source*: Authors’ original data

*n*, given as means of number.

### Association between acceptance of Intimate Partner Violence, actual Intimate Partner Violence inquiry and sociodemographic factors

The marital status (χ^2^ [2] = 9, 49, *p* < 0.01) and employment status (χ^2^ [1] = 4, 4, *p* < 0.05) are indicated in Table 3. There was no significant association between acceptance for screening and the other demographic variables.

**TABLE 3:** Association between acceptance for Intimate Partner Violence inquiry, actual Intimate Partner Violence inquiry and sociodemographic characteristics of participants.

Sociodemographic characteristics of participants	Additional characteristics	Acceptance	Actual screening	Variables			
		
		*N*	*n*†	%	*N*	*n*‡	%
Profession	Housewife	167	132	79.0	175	16	9.1
	Others§	158	130	82.3	173	16	9.2
Marital status	Married	332	270	81.3	348	33	9.5
	Single	100	77	77.0	110	0	–
	Divorced or Separated	16	8	50.0	21	0	–
Religion	Islam	304	247	81.2	330	27	8.2
	Others¶	144	108	75.0	149	6	4.0
Ethnicity	Hausa or Fulani	279	227	81.4	299	27	9.0
	Others††	168	127	75.6	179	6	3.4
Marital situation	Monogamous	186	147	79.0	190	14	7.3
	Polygamous	114	94	82.5	122	16	13.1
Employment	Employed	115	99	86.1	123	10	8.1
	Not employed	324	249	76.9	347	22	6.3
Literacy level	Cannot read at all	78	62	79.5	80	6	7.5
	Able to read	359	283	78.8	389	26	6.7
Educational level	No education	71	58	81.7	74	4	5.4
	Primary	31	24	77.4	36	4	11.1
	Secondary	173	135	78.0	181	5	2.7
	Post-secondary	166	132	79.5	181	18	9.9
	Islamic or Quranic	4	3	75.0	4	2	50.0

*Source*: Authors’ original data

*N*, total number in the category

† *n*, number of participants accepting

‡ *n*, number of participants actually screened.

§ Gainfully employed professionals including students.

¶ Christianity and other minority religions.

†† Yoruba, Ibo and other ethnic groups.

Ethnicity (χ^2^ [1] = 5,6, *p* < 0.05) was associated with being probed about IPV in healthcare (Table 3); in other words, participants of Hausa or Fulani ethnic group were more often probed about the possibility of IPV in healthcare than the other ethnic groups together (i.e. migrant ethnic groups in the region). A trend was observed regarding the association between probing about IPV in healthcare and religion (χ^2^ [1] = 2.8, *p* < 0.09), where Muslim participants seemed more likely to have been probed on IPV than other religions combined. There was no statistical association between being probed about IPV in healthcare and other demographic factors.

### Association between actual Intimate Partner Violence inquiry and satisfaction with care

There was an association (Table 4) between IPV-probing in healthcare and satisfaction with nursing staff (*t* [469] = 4.74; *p* < 0.001), contact (*t* [466] =3, 51; *p* < 0.001) and social support (*t* [462] =4.19; *p* < 0.001). Participants who had been probed on IPV in their latest contact expressed on average higher satisfaction in these regards than peers who had not been probed on IPV.

**TABLE 4:** Association between actual Intimate Partner Violence inquiry and satisfaction with care (nursing staff, contact, and social support).

Screened	Nursing staff				Contact				Social support			
		
	*n*	Mean	s.d.	*P*	*n*	Mean	s.d.	*P*	*n*	Mean	s.d.	*P*
Yes	33	11.3	1.3	0.001	32	10.8	2.1	0.001	31	14.9	2.7	0.001
No	438	9.5	2.2	0.001	436	9.4	2.2	0.001	433	12.7	2.7	0.001

**Total**	**471**	**9.6**	**2.2**	**–**	**468**	**9.5**	**2.2**	**–**	**464**	**12.9**	**2.8**	**–**

*Source*: Authors’ original data

*n*, given as means of number; s.d., standard deviation; *P*, given as *P*-value.

## Discussion

The current paper studied women's preferences for screening, actual inquiry of Intimate Partner Violence (IPV) in Kano, Nigeria. The majority of women (76%) in our sample opted for IPV inquiry in healthcare. These figures are comparable to those observed in high income settings. In their Swedish sample, Stenson et al.,^3^ found that 80% of women in their study sample to opt for IPV inquiry. In a sample from New Zealand, up to 97% of clients opted for screening.^37^ Although encouraging, the findings were rather surprising. Studies from the region have abundantly found women to be reluctant to disclose abuse.^18, 24, 25, 29, 38^ Thus an important implication of these results is that women find inquiry of IPV acceptable, but for some reason may not be in position yet to disclose if asked. Societal norms and laws that are gender restrictive may account for the failure to disclose. Further analysis of this discrepancy is warranted to disclose where, for some reasons, women decline disclosure of violence and hence did not accept inquiry on abuse.

Married women were more willing to accept IPV screening than were women with other civil status. Married participants are classified as ‘currently in a relationship’ and are consequently more likely to be affected by IPV and to find such inquiry warranted. These findings could be a reflection of such circumstances. Unemployment was also significantly associated with unacceptable preference for screening for IPV, a finding that concurs with previous research.^11, 26^ Women at the lower bracket of the socio-economic hierarchy are more likely to justify violence and may not readily disclose abuse. Their unemployed status may hinder such women from accepting screening because they are more likely to be economically dependent on their abusive partners.^8^

Our findings revealed that only 7% of the clients were probed for the possibility of IPV by care providers, corroborating studies in high income countries where barely 10% of HCPs screen for IPV.^2, 39, 40^ This has important implications for the initiation of a screening campaign in the hospital. Education and supervision of healthcare providers on routine screening for IPV has been shown to improve actual screening in some settings.^33, 41^ Our results demonstrated further that ethnic and religious majorities (Hausa or Fulani and Muslims) were more likely to be screened, suggesting demographic inequalities in the access to care (in this case access to screening). Recent studies from the same hospital suggest that care providers from ethnic and religious majorities were more likely to screen.^42^ These findings together suggest that the migrant populations in the region may be denied care to a higher degree by their indigenous peers.

Our results revealed that clients who had been probed about IPV, exhibited higher *satisfaction with care* than peers who had not been probed, corroborating recent work in the field.^43^ Screening may offer easy access to support services and management of immediate and later trauma of patients, thereby enhancing satisfaction.^37, 44^

## Conclusion

In summary, this study provided new insight on the acceptance of screening, actual screening and *satisfaction with care*. Acceptance for screening was exceptionally high in a societal context where women's acceptance of IPV is high.^16^ As expected, few women had actually been probed about violence in their contact with care providers. Acceptance for screening was associated with being married and being employed. Actual screening was associated with ethnicity and religion, with ethnic and religious majorities more likely to be screened. Finally, screening for IPV seemed to improve *satisfaction with care*. The findings demonstrate the need for the adaptation of a screening protocol at the hospital, sensitive however to detecting IPV amongst all ethnic and religious groups. The findings also have implications for further education of socio-economically disadvantaged women on the benefits of screening.

## References

[1] Violence against Women: A Priority Health Issue Geneva: World Health Organization; 1997.

[2] Waalen J, Goodwin MM, Spitz AM, Petersen R, Saltzman LE. Screening for Intimate Partner Violence by Health Care Providers: barriers and Interventions. Am J Prev. Med. 2000;19:230–237. doi:10.1016/S0749-3797(00)00229-4 11064226

[3] Stenson K, Saarinen H, Heimer G, Sidenvall B. Women's attitudes to being asked about exposure to violence. Midwifery. 2001;17(1):2–10. doi:10.1054/midw.2000.0241, PMid:11207100

[4] Battaglia TA, Finley E, Liebschutz JM. Survivors of Intimate Partner Violence speak out–trust in the patient-provider relationship. J Gen Intern Med. 2003;18:617–623. doi:10.1046/j.1525-1497.2003.21013.x, PMid:, PMCid:12911643 PMC1494898

[5] Zeitler MS, Paine AD, Breitbart V et al Attitudes about Intimate Partner Violence Screening among an ethnically diverse sample of young women. J Adolesc Health. 2006;39:119.e1–119.e8. 16781970 10.1016/j.jadohealth.2005.09.004

[6] Rickert V, Davison L, Breitbart V. A Randomized Trial of Screening for Relationship Violence in Young Women. J Adolesc Health. 2009;45(2):163–170. doi:10.1016/j.jadohealth.2008.12.012, PMid:19628143

[7] Jejeebhoy S. Wife-beating in rural India: a husband's right? Econ Polit Wkly. 1998;33:855–862.

[8] Fawole OI, Aderonmu AL, Fawole AO. Intimate Partner Abuse: Wife Beating among Civil Servants in Ibadan, Nigeria. Afr J Repr Health. 2005;9:54–64. doi:10.2307/3583462, PMid:16485586

[9] Lawoko S. Attitudes towards wife abuse: A comparative study of men and women in Kenya In: Health knowledge, Attitudes and Practices. Ed. Eddington PI, Mastolli UV Nova Science; 2008.

[10] Haj-Yahia MM. Beliefs of Jordanian women about wife-beating. Psychol Women Q. 2002;26:282–291. doi:10.1111/1471-6402.t01-1-00067

[11] Hindin MJ. Understanding women's attitudes towards wife beating in Zimbabwe. Bull World Health Organ. 2003;81:501–508. 12973642 PMC2572507

[12] Rani M, Bonu S, Diop-Sidibe N. An empirical investigation of attitudes towards wife-beating among men and women in seven sub-Saharan African countries. Afr J Repr Health. 2004;8:116–136. doi:10.2307/3583398, PMid:17348330

[13] Arias I, Johnson P. Evaluation of physical aggression among intimate dyads. J Interpersonal Viol. 1989;4:298–307. doi:10.1177/088626089004003004

[14] Greenblat CS. 'Don't hit your wife … unless …’, Preliminary findings on normative support for the use of physical force by husbands. Victimology. 1985;10:221–241.

[15] Kazungu M, Chewe PM. Violence against women. Zambia Demographic and Health Survey, Final report; 2002.

[16] Uthman OA, Moradi T, Lawoko S. The independent contribution of individual-, neighbourhood-, and country-level socioeconomic position on attitudes towards intimate partner violence against women in Sub-Saharan Africa: A multilevel model of direct and moderating effects. Soc Sci Med. 2009;68(10):1801–1809. doi:10.1016/j.socscimed.2009.02.045 19303687

[17] Domestic Violence, Nigeria 2000 The Penal Code of Northern Nigeria. Women Action16.3 [homepage on the Internet]. c2000 [updated 2000 March; cited 2009 July 19]. Available from http://www.equalitynow.org/english/wan/beijing5/beijing5_violence_en.html#domestic

[18] Nash ST. Through black eyes African American women's constructions of their experiences with intimate male partner violence. VAW. 2005;11:1420–1440. 10.1177/1077801205280272 16204732

[19] McCauley J, Yurk RA, Jenckes MW, Ford DE. Inside 'Pandora's Box’ abused women's experiences with clinicians and health services. JGen Intern Med. 1998;13:549–555. doi:10.1046/j.1525-1497.1998.00166.x, PMid:, PMCid:9734792 PMC1497000

[20] Murdaugh C, Hunt S, Sowell R, Santana I. Domestic violence in Hispanics in the Southeastern United States: a survey and needs analysis. J Fam Viol 2004;19:107–115. doi:10.1023/B:JOFV.0000019841.58748.51

[21] Fugate M, Lands L, Riordan K, Naureckas S, Engel B. Barriers to domestic violence help seeking. VAW. 2005;11:290–310. 10.1177/1077801204271959 16043551

[22] Morrison KE, Luchok KJ, Richter DL, Parra-Medina D. Factors influencing help-seeking from informal networks among African American victims of intimate partner violence. J Interpersonal Viol. 2006;21:1493–1511. doi:10.1177/0886260506293484, PMid:17057164

[23] Krishnan SP, Hilbert JC, Van Leeuwen D. Domestic violence and help-seeking behaviors among rural women: results form a shelter-based study. Fam Community Health. 2001;24:28–38.PMid:11275569 10.1097/00003727-200104000-00006

[24] Dowd MD, Kennedy C, Knapp JF, Stallbaumer-Rouyer J. Mothers’ and Health care Providers’ Perspectives on screening for Intimate Partner Violence in a Paediatric Emergency Department. Arch Paed Adolesc Med. 2002;156:794–799.PMid:12144370 10.1001/archpedi.156.8.794

[25] Gerbert B, Abercrombie P, Caspers N, Love C, Bronstone A. How healthcare providers help battered women: survivor's perspective. Women and Health. 1999;29:115–135. doi:10.1300/J013v29n03_08 10466514

[26] Naved RT, Azim S, Bhuiya A, Persson IA. Physical violence by husbands: magnitude, disclosure and help-seeking behaviour of women in Bangladesh. Soc Sci Med. 2006;62:2917–2929. doi:10.1016/j.socscimed.2005.12.001 16426717

[27] Gonzales-Brenes M. Domestic violence and household decision-making: evidence from East Africa University of California, Berkerly; 2004

[28] Lawoko S. Factors associated with Attitudes towards Intimate Partner Violence: A study of Women in Zambia. Viol Vict. 2006;21:645–656. doi:10.1891/vivi.21.5.645, PMid:17022355

[29] Okenwa LE, Lawoko S, Jansson B. Exposure to Intimate Partner Violence Amongst Women of Reproductive Age in Lagos, Nigeria: Prevalence and Predictors. JFam Viol. 2009;24:517–530. doi:10.1007/s10896-009-9250-7

[30] Soglin LF, Bauchat J, Soglin DF, Martin GJ. Detection of Intimate Partner Violence in General Medicine Practice. J interpersonal Viol. 2009;24:338–348. 10.1177/0886260508316481 18667690

[31] Ntaganira J, Muula AS, Masaisa F, Dusabeyezu F, Siziya S, Rudatsikira E. Intimate Partner Violence among pregnant women in Rwanda. BMC Women's Health. 2008;8:17. doi:10.1186/1472-6874-8-17, PMid:, PMCid:18847476 PMC2570659

[32] Phelan MB. Screening for Intimate partner violence in medical settings. Trauma Viol Abuse. 2007;8:199–213. doi:10.1177/1524838007301221 17545574

[33] Davidson LL, Grisso JA, Garcia-Moreno C, Garcia J, King VJ, Marchant S. Training programs for healthcare professionals in domestic violence. J Women's health Gend Based Med. 2001;10:953–969. doi:10.1089/152460901317193530 11788106

[34] Ilika AL, Okonkwo PI, Adogu P. Intimate Partner Violence among Women of Childbearing Age in a Primary Health Care Centre in Nigeria. Afr J Repr Health. 2002;6:53–58. doi:10.2307/3583257, PMid:12685409

[35] Koenig MA, Lutalo T, Zhao F, et al. Domestic violence in rural Uganda: evidence from a community-based study. Bull World Health Organ. 2003;81:53–60.PMid:, PMCid:12640477 PMC2572313

[36] Arnetz JE, Arnetz BB. The development and application of a patient satisfaction measurement system for hospital-wide quality improvement. Int J Qual Health Care. 1996;8:555–566. doi:10.1016/S1353-4505(96)00073-7 9007605

[37] Koziol-McLain J, Giddings L, Rameka M, Fyfe E. Intimate partner violence screening and brief intervention: experiences of women in two New Zealand Health Care Settings. J Midwifery Women's Health. 2008;53:504–510. doi:10.1016/j.jmwh.2008.06.002 18984506

[38] Kershner M, Anderson JE. Barriers to disclosure of abuse among rural women. Minnesota Med J. 2002;85:32–37. 11915526

[39] Erikson MJ, Hill TD, Siegal RM. Barriers to domestic Violence screening in the Padiatric Setting. Pediatrics. 2001;108:98–102. doi:10.1542/peds.108.1.98, PMid:11433060

[40] Roelens K, Verstraelen H, Van Egmond K, Temmerman M. A knowledge, attitudes, and practice survey among obstetrician-gynaecologists on intimate partner violence in Flanders, Belgium. BMC Public Health. 2006;26:238. doi:10.1186/1471-2458-6-238, PMid:, PMCid:17002786 PMC1635712

[41] Friedman LS, Samet JH, Roberts MS, Hudlin M, P. Inquiry about victimization experiences. A survey of patient preferences and physician practices. Arch Intern Med. 1992;152:1186–1190. doi:10.1001/archinte.152.6.1186, PMid:1599346

[42] John IA, Lawoko S, Svanström L, Mohammed AZ. Health Care Providers readiness to screen for Intimate Partner Violence in Northern Nigeria. Viol Vict. 2010;25(5):689–704. doi:10.1891/0886-6708.25.5.689, PMid: 21061873

[43] Manchester AA. Asking about abuse and assessing for family violence–part of nurses’ caring role. Nursing New Zealand. 2007;13(10):10–11. PMid:18084964

[44] Taket A, Nurse J, Smith K, et al. Routinely asking women about domestic violence in health settings. BMJ. 2003;327:673–6. doi:10.1136/bmj.327.7416.673, PMid:, PMCid:14500444 PMC196400

